# Modulation of the p38 MAPK Pathway by Anisomycin Promotes Ferroptosis of Hepatocellular Carcinoma through Phosphorylation of H3S10

**DOI:** 10.1155/2022/6986445

**Published:** 2022-11-24

**Authors:** Wei Chen, Wenjing Yang, Chunyan Zhang, Te Liu, Jie Zhu, Hao Wang, Tong Li, Anli Jin, Lin Ding, Jingrong Xian, Tongtong Tian, Baishen Pan, Wei Guo, Beili Wang

**Affiliations:** ^1^Department of Laboratory Medicine, Zhongshan Hospital, Fudan University, Shanghai, China; ^2^Department of Laboratory Medicine, Xiamen Branch, Zhongshan Hospital, Fudan University, Xiamen, China; ^3^Shanghai Geriatric Institute of Chinese Medicine, Shanghai University of Traditional Chinese Medicine, Shanghai, China; ^4^Department of Laboratory Medicine, Wusong Branch, Zhongshan Hospital, Fudan University, Shanghai, China; ^5^Cancer Center, Shanghai Zhongshan Hospital, Fudan University, Shanghai, China

## Abstract

Hepatocellular carcinoma (HCC) is a prevalent malignant tumor worldwide. Ferroptosis is emerging as an effective target for tumor treatment as it has been shown to potentiate cell death in some malignancies. However, it remains unclear whether histone phosphorylation events, an epigenetic mechanism that regulates transcriptional expression, are involved in ferroptosis. Our study found that supplementation with anisomycin, an agonist of p38 mitogen-activated protein kinase (MAPK), induced ferroptosis in HCC cells, and the phosphorylation of histone H3 on serine 10 (p-H3S10) was participated in anisomycin-induced ferroptosis. To investigate the anticancer effects of anisomycin-activated p38 MAPK in HCC, we analyzed cell viability, colony formation, cell death, and cell migration in Hep3B and HCCLM3 cells. The results showed that anisomycin could significantly suppress HCC cell colony formation and migration and induce HCC cell death. The hallmarks of ferroptosis, such as abnormal accumulation of iron and elevated levels of lipid peroxidation and malondialdehyde, were detected to confirm the ability of anisomycin to promote ferroptosis. Furthermore, coincubation with SB203580, an inhibitor of activated p38 MAPK, partially rescued anisomycin-induced ferroptosis. And the levels of p-p38 MAPK and p-H3S10 were successively increased by anisomycin treatment. The relationship between p-H3S10 and ferroptosis was revealed by ChIP sequencing. The reverse transcription PCR and immunofluorescence results showed that NCOA4 was upregulated both in mRNA and protein levels after anisomycin treatment. And by C11-BODIPY staining, we found that anisomycin-induced lipid reactive oxygen species was reduced after NCOA4 knockdown. In conclusion, the anisomycin-activated p38 MAPK promoted ferroptosis of HCC cells through H3S10 phosphorylation.

## 1. Introduction

Hepatocellular carcinoma (HCC) is the third leading cause of cancer-related deaths worldwide [1]. Numerous drugs have been developed to improve the outcomes of patients with HCC [2]. The targets of these drugs include CRAF, BRAF, EGFR, and VEGFR. However, until now, the overall survival rate of HCC patients is still limited, increasing the urgent demand for the exploration of additional targets and drugs [3].

The p38 mitogen-activated protein kinase (MAPK) is a stress-activated protein kinase, which could be activated by environmental and intracellular stresses and then stimulate downstream proteins, such as MKK3/6, MSK1/2, or WHIP1 [4]. Some researchers have reported the anticancer effects of p38 MAPK activation, showing its promising potential in cancer treatment. Yao et al. [5] found that activation of p38 MAPK could promote the phosphorylation of heat shock protein 27 and induce apoptosis in lung squamous cell carcinoma. Furthermore, Zhang et al. [6] found that fenretinide-stimulated p38 MAPK activation could reduce the activation of myosin light chain kinase, thus inhibiting the proliferation and migration of HepG2 cells from human liver cancer. Recently, some studies have found that the p38 MAPK signaling pathway could promote ferroptosis in endometrial stromal cells [7], osteoblasts [8], and cancer cells [9, 10]. More interestingly, noncoding RNAs have also been reported to be involved in the axis of p38-ferroptosis [11, 12]. Moreover, an agonist of p38 MAPK, anisomycin [13], has also shown its potential value in medication, as it could significantly induce cell death in acute lymphoblastic leukemia, melanoma, and glioma [14–18]. Kim et al. [19] described that anisomycin could exert both direct killing effects and immunotherapeutic effects mediated by natural killer cells (NK) in HCC. However, no studies have demonstrated the relationship between anisomycin and ferroptosis.

Histones, known to be part of nucleosomes, undergo multiple types of posttranslational modifications (PTM), such as acetylation, methylation, ubiquitination, phosphorylation, and SUMOylation, and participate in the regulation of chromatin condensation and DNA accessibility [20–22]. Several studies have found that p38 MAPK could promote phosphorylation of histone H3S10/S28 [23–25]. Phosphorylated histone H3 usually modulates chromatin structure to enhance DNA accessibility and subsequently promote transcriptional activation or coordinate another histone PTM [26–28]. In addition, phosphorylation of histone H3 is involved in different biological activities of cancer. For example, H3 phosphorylation through the p38 MAPK signaling pathway has been demonstrated to participate casticin-induced cytocidal effects against the human promyelocytic cell line HL-60 [29].

Ferroptosis, an iron-dependent form of nonapoptotic cell death [30], is characterized by iron overload and lipid peroxidation [31, 32]. The canonical ferroptosis induction pathway is activated mainly directly or indirectly by inactivating the main protective mechanism against peroxidation damage, especially glutathione peroxidase 4 (GPX4). Meanwhile, noncanonical ferroptosis induction refers to ferroptosis that is initiated by the accumulation of labile iron pool (LIP), small pools of iron mainly in the form of free ferrous iron (Fe^2+^) [31, 33]. It is well known that maintaining iron homeostasis is important for cell death and human diseases [34], including noncanonical ferroptosis induction. Increased LIP can directly promote the Fenton reactions and can further increase lipid peroxidation. As one of the inducers of ferroptosis, nuclear receptor coactivator 4 (NCOA4) has been widely reported to regulate ferroptosis mainly by increasing LIP [35–39]. Furthermore, many studies have demonstrated that drug-induced ferroptosis was reduced after NCOA4 knockdown [40–42], and several studies have also demonstrated that drug-induced ferroptosis was increased after NCOA4 overexpression [43]. In human liver cancer cell HepG2, the study by Hattori et al. [44] had found that the activation of the ASK1-p38 pathway was involved in cold stress-induced ferroptosis, although the mechanism has not yet been clarified. To date, the association of phosphorylated H3S10/28 and ferroptosis in HCC and the underlying mechanisms have not been reported.

In this study, we used anisomycin to activate p38 MAPK and investigated whether and how activation of p38 MAPK participates in the killing of HCC cells. Furthermore, we also explored whether histone H3 phosphorylation is involved in ferroptosis promoted by anisomycin-induced activation of p38 MAPK.

## 2. Materials and Methods

### 2.1. Cell Culture and Transfection

The HCC cell lines, HCCLM3 and Hep3B, were obtained from Liver Cancer Institute, Zhongshan Hospital, Fudan University (Shanghai, China). All cells were cultured in Dulbecco's Modified Eagle Medium (DMEM, Gibco, USA) supplemented with 10% fetal bovine serum (FBS; BioSun, China) and 1% antibiotics (100 U/mL penicillin G and 100 mg/mL streptomycin, Gibco, USA). Cells were cultured at 37°C in an incubator (Thermo Fisher, USA) with a humidified atmosphere containing 5% CO_2_.

The plasmid or small interfering RNA (siRNA) oligos were transfected into cells by jetPRIME reagent (Polyplus, France) when cells reached 70% confluency. The plasmid of NCOA4 was purchased from GenePharma (Shanghai, China), and they used pEX-6 (pGCMV/MCS/RFP/Neo) as the carrier vector for NCOA4 cDNA. Gene-specific and negative control (NC) siRNAs were synthesized by GenePharma (Shanghai, China). The siRNA sequence for NCOA4#1 was 5′-ACTCTTGTTTATCGAAGTATA-3′ and for NCOA4#2 was 5′-CTCTTATTCCAGTCCTATAAT-3′ [40]. RT-PCR was used to test NCOA4 knockdown and overexpression in Hep3B and HCCLM3.

### 2.2. Chemicals and Antibodies

Anisomycin, SB203580 [45], SP600125, and ferrostatin-1 [46] were purchased from MCE (Shanghai, China) and dissolved in dimethyl sulfoxide (DMSO). Cells were treated with anisomycin at the indicated concentration or coincubated with SB203580 (20 *μ*M) or ferrostatin-1 (4 *μ*M). Commercially available antibodies were used in this study. The anti-tubulin antibody was obtained from InTech (Shanghai, China), while anti-c-Myc (#18583), CD133 (#64326), Nanog (#4903), EpCAM (#2929), caspase-3 (#14220), Bcl-2 (#15071), Bax (#5023), N-cadherin (#13116), E-cadherin (#3195), vimentin (#5741), *α*-smooth muscle actin (*α*-SMA, #19245), p38 MAPK (#8690), phospho-p38 MAPK (p-p38 MAPK, #9216), histone H3 (#4499), phospho-histone H3 (Ser10) (p-H3S10, #53348), phospho-histone H3 (Ser28) (p-H3S28, #9713), NRF2 (#12721), SLC7A11 (#12691), FTH1 (#4393), and NCOA4 (#66849) antibodies were purchased from Cell Signaling Technology (Boston, USA). Anti-CD24 (ab31622) antibody was provided by Abcam (Cambridge, UK).

### 2.3. Cell Viability Assay

Cell viability was evaluated using the Cell Counting Kit-8 (CCK-8; Beyotime, China) according to the manufacturer's instructions. Briefly, cells (1 × 10^4^ cells/well/100 *μ*L) were seeded in a 96-well plate and treated with anisomycin at different concentrations for 24 h. Then, 10 *μ*L of CCK-8 solution was added to each well, and the plates were incubated at 37°C for another 2 h. The absorbance values of the samples were evaluated at 450 nm using a microplate reader. Experiments were performed in triplicate.

### 2.4. Colony Formation Assay

Cells were plated at a density of 4 × 10^4^ cells per well in 6-well plates and allowed to attach. The following day, cells were treated with DMSO, anisomycin, or SB203580 plus anisomycin for 12 h. Then, the medium was replaced with DMEM supplemented with 10% FBS. The medium was changed every 3 days for 2 consecutive weeks. Finally, colonies were stained with crystal violet staining solution (Beyotime, China) for 15 min after fixation with 4% paraformaldehyde for 10 min. The stained colonies were washed with phosphate buffer saline (PBS) and counted using the ImageJ software. Experiments were performed in triplicate.

### 2.5. Cell Death Detection

Cell death was evaluated using an apoptosis detection kit purchased from BD Biosciences (San Jose, USA). Cells were harvested, stained with annexin V antibody and 7AAD, and then analyzed with a FACSAria™ II flow cytometer (BD Biosciences, USA). The flow cytometry results were further analyzed with the FlowJo 10.1 software (Tree Star, Inc., USA). The calculation of cell death rate was (1, the proportion of viable cells (cells in Q4)). Experiments were performed in triplicate.

### 2.6. Lipid Reactive Oxygen Species (Lipid-ROS) Levels

The accumulation of lipid-ROS is a major feature of ferroptosis and can be used to infer the degree of ferroptosis [47]. Cells were treated with DMSO, anisomycin, or SB203580 plus anisomycin for 24 h or 12 h. Then, cells were incubated with 3 *μ*M C11-BODIPY 581/591 (Invitrogen, USA) in serum-free DMEM medium for 30 min at 37°C in an incubator. Afterwards, cells were washed three times with serum-free medium, trypsinized, and resuspended in PBS before detected by flow cytometry. Lipid-ROS levels were evaluated by the fluorescence intensity of FITC channel, and the mean fluorescence intensities (MFI) of FITC were calculated for each sample. Experiments were performed in triplicate.

### 2.7. Wound-Healing Assay

When Hep3B and HCCLM3 cells reached 100% confluency in 24-well plates, cells were wounded with a sterile 10 *μ*L pipette tip in the cell monolayers and washed with serum-free medium to remove detached cells. Cells were then treated with DMSO, anisomycin, or SB203580 plus anisomycin at the indicated concentration for 12 h. The medium was then replaced with DMEM supplemented with 2% FBS, and cells were cultured for 48 h. The wound gap images were taken using a microscope (Olympus, Japan). Healing areas were calculated using the ImageJ software. Experiments were performed in triplicate.

### 2.8. Transwell Assay

Cells were treated with DMSO, anisomycin, or SB203580 plus anisomycin for 12 h before seeding (5 × 10^4^) and cultured in the upper chamber (24-well Transwell chambers, 8 *μ*m pore size, Corning, USA) with serum-free DMEM, while serum-containing (10% FBS) DMEM was added to the lower chamber. After 48 h incubation at 37°C, cells were fixed with 4% paraformaldehyde and stained with crystal violet staining solution (Beyotime, China). Cells stained were imaged using a microscope (Olympus, Japan). Experiments were performed in triplicate.

### 2.9. Western Blotting

Cells were washed with PBS, collected, resuspended in RIPA lysis buffer (Beyotime, China), and kept on ice for 30 min. The suspension was vibrated vigorously every 5 min. Sonication was required. Cell extracts were obtained by centrifugation at 12,000 × *g* for 10 min at 4°C. The protein concentration was determined using a bicinchoninic acid (BCA) protein assay kit (Thermo Fisher, USA). The denatured proteins were separated by sodium dodecyl sulfate-polyacrylamide gel electrophoresis (SDS-PAGE) and electrotransferred onto polyvinylidene difluoride (PVDF) membranes (Merck Millipore, Germany). The membranes were blocked with QuickBlock blocking buffer (Beyotime, China), incubated with diluted primary antibodies (1 : 1000) for 12 h at 4°C, and washed three times with TBST for 10 min. The membranes were then incubated with HRP conjugated secondary antibodies (1 : 2000) for 2 h at room temperature. Hybridization was detected using enhanced chemiluminescence reagents (ECL; Beyotime, China) after three rinses with TBST for 10 min. Image Lab 4.1 software and ImageJ software were used to quantify western blot bands.

### 2.10. Immunofluorescence Analysis

Cells were fixed in 4% paraformaldehyde for 15 min at room temperature and permeabilized with 0.1% Triton X-100 (Beyotime, China) for 10 min at 4°C. Cells were saturated with TBST containing 2% bovine serum albumin (BSA, Merck Sigma, Germany) for 1 h at room temperature before incubation with primary antibodies against p-p38 MAPK (1 : 200), p-H3S10 (1 : 100), or NCOA4 (1 : 400) overnight at 4°C. Subsequently, cells were incubated with a secondary antibody labeled Alexa Fluor 488 or 594 (Invitrogen, USA) (1 : 2000). Finally, cells were incubated with DAPI (5 *μ*l DAPI in 200 *μ*l TBST) for 10 min. After each antibody incubation step, cells were washed three times for 5 min with TBST. Fluorescence signals were observed using an Olympus Fluoview microscope (Olympus Corp, Japan).

### 2.11. Chromatin Immunoprecipitation- (ChIP-) Sequencing

ChIP-sequencing was outsourced to Kangcheng Biotechnology (Shanghai, China). Briefly, the p-H3S10 antibody was used for immunoprecipitation. A total of 10 ng of DNA samples were prepared and blunt-ended. Then, a dA base was added to the 3′ end of each strand, and genomic adapters were ligated to the DNA fragments. Subsequently, PCR amplification was performed to enrich the ligated fragments, and ~200–1500 bp fragments < 200-1500 bp were selected using AMPure XP beads. The libraries were then sequenced on the Illumina NovaSeq 6000 instrument following the NovaSeq 6000 S4 reagent kit protocol (300 cycles). The reads were aligned to the human genome (UCSC HG19) using the BOWTIE software (V2.2.7). Aligned reads were used to call the peaks of the ChIP regions using MACS V1.4.2. A *P* value threshold of 10^−3^ was used to select differentially enriched peaks.

### 2.12. Bioinformatics

The R packages “clusterProfiler,” “org.Hs.eg.db,” “enrichplot,” and “ggplot2” were used in R 4.0.3 software to perform functional enrichment analyzes of Gene Ontology (GO) and Kyoto Encyclopedia of Genes and Genomes (KEGG). Furthermore, the STRING 11.5 online tool (https://cn.string-db.org/) was used to find protein-protein interactions (PPI) between the selected genes with a confidence filter (interaction score > 0.150).

### 2.13. Iron Assay

Intracellular iron was assessed using an iron colorimetric assay kit (APPLYGEN, China) according to the manufacturer's instructions. The results were assessed at 550 nm using a microplate reader and normalized to protein concentration. Experiments were performed in triplicate.

### 2.14. Lipid Peroxidation (LPO) Assay

The Lipid Peroxidation Assay Kit (Nanjing Jiancheng Institute of Bioengineering, China) was used to measure LPO levels in HCC cells. The results were evaluated at 586 nm using a microplate reader and normalized to protein concentration. All procedures were performed in full accordance with the manufacturer's instructions. Experiments were performed in triplicate.

### 2.15. Malondialdehyde Assay

The relative concentration of malondialdehyde (MDA) in cell lysates was evaluated using a lipid peroxidation MDA assay kit (Beyotime, China) according to the manufacturer's instructions. The MDA level was determined by a microplate reader at 532 nm and normalized to protein concentration. Experiments were performed in triplicate.

### 2.16. Quantitative Reverse Transcription Polymerase Chain Reaction (qRT-PCR)

TRIzol reagent (Invitrogen, USA) was used to extract total RNA from HCC cells. RNA was reverse transcribed to cDNA using GoScript™ Reverse Transcription Mix (Promega, USA) according to the manufacturer's instructions. The cDNA was then detected using the GoTaq® qPCR Master Mix reagent (Promega, USA). The PCR program included denaturation at 94°C for 2 min, followed by 40 cycles of 94°C for 30 s, 56°C for 30 s, 72°C for 30 s, and finally a 5 min elongation at 72°C. The sequences of primers used are listed in Supplementary Table 1. *β*-Actin was included as the internal control to assess the relative expression of the genes. The relative level of mRNA expression was calculated using 2^-△△Ct^. Experiments were performed in triplicate.

### 2.17. Statistical Analysis

All statistical analyses were performed using the GraphPad Prism software 7.0 (GraphPad Software Inc., USA), and data were displayed as mean ± standard deviation (SD). The means of the groups were compared using the Student's *t*-test for two independent groups. When the variances were uneven (*P* < 0.1), Welch's *t*-test was used. All *P* values were two-tailed, and *P* < 0.05 was considered statistically significant.

## 3. Results

### 3.1. Anisomycin Induced HCC Cell Death through the p38 MAPK Signaling Pathway

**Figure 1:**
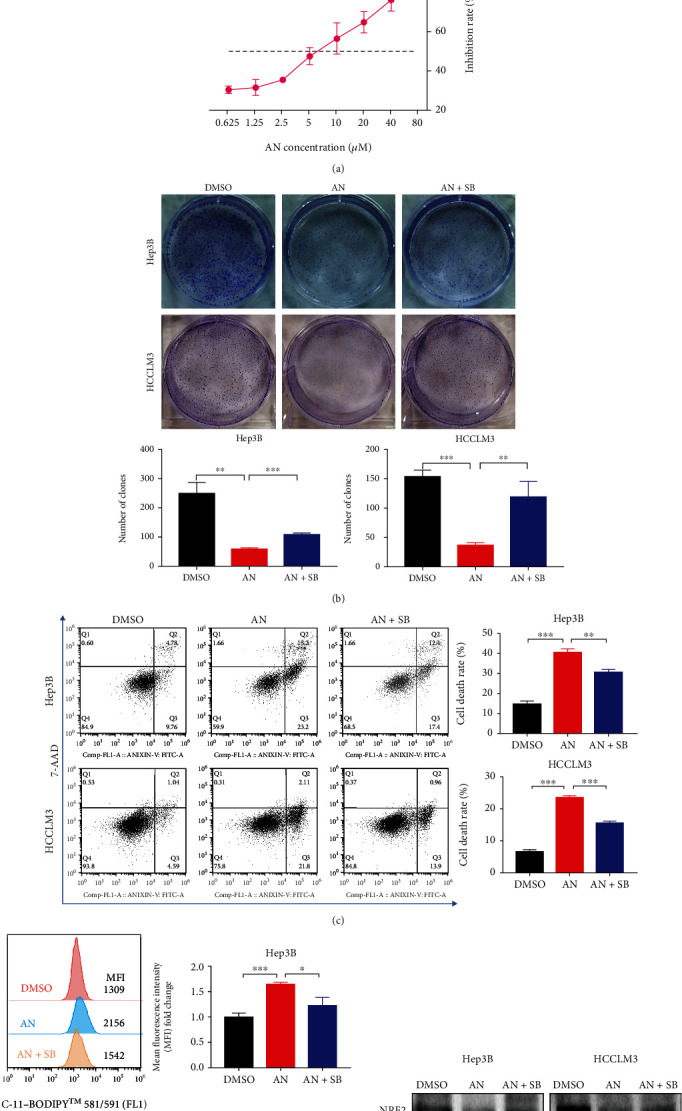
Anisomycin (AN) induced HCC cell death through the p38 MAPK pathway. (a) The inhibition rate of Hep3B and HCCLM3 cell proliferation after 24 h treatment with AN. (b) Colony formation of HCC cells under AN treatment. SB203580 (SB), an inhibitor of phosphorylated p38, was used to rescue cells from AN treatment. (c) Cell death rate of HCC cells induced by AN (2.5 *μ*M for Hep3B or 5 *μ*M for HCCLM3). (d) The flow cytometry results indicated the accumulation of intracellular lipid reactive oxygen species after 24 h AN treatment. *N* = 3; ^∗^*P* < 0.05, ^∗∗^*P* < 0.01, and ^∗∗∗^*P* < 0.001. (e) The expression levels of ferroptosis-related proteins were analyzed by western blotting after 24 h AN treatment.

Expression levels of p38 MAPK and p-p38 MAPK in different HCC cell lines were evaluated by western blotting (Supplementary Figure 1A). Hep3B and HCCLM3, two cell lines with weak phosphorylation of p38 MAPK, were chosen for subsequent experiments of activation of p38 MAPK. Anisomycin treatment was applied to the two cell lines to investigate the anticancer effects of p38 MAPK activation on HCC cells [14–16]. The results of 24 h anisomycin treatment showed that anisomycin inhibited the proliferation of HCC cells in a dose-dependent manner (Figure 1(a)). When the inhibition rate reached 50%, the anisomycin concentrations for Hep3B and HCCLM3 were approximately 2.5 *μ*M and 5 *μ*M, respectively. The indicated concentrations of anisomycin (2.5 *μ*M for Hep3B and 5 *μ*M for HCCLM3) were selected for the following experiments. Besides, we performed CCK8 analysis for all 6 cell lines in Supplementary Figure 1A after treatment with different concentrations of anisomycin and integrated all curves. The results showed that low p-p38/p38 ratio cells (Hep3B, HCCLM3, and SK-Hep-1) required lower concentrations of anisomycin than high p-p38/p38 ratio cells (MHCC-97L, MHCC-97H, and HepG2) to reach 50% inhibition rate (Supplementary Figure 1B).

The colony formation assay and cell death detection were performed to further assess the anticancer effects of anisomycin on HCC cells. We shortened the treatment time to 12 h for cell functional assays (colony formation assay, wound-healing assay, and transwell assay) to reduce interference from cell death with experimental results. The number of clones was substantially reduced for cells treated with anisomycin for 12 h, and the cell death rate was substantially increased for cells treated with anisomycin for 24 h. Meanwhile, the anticancer effects of anisomycin were attenuated by SB203580, an inhibitor of activated p38 MAPK (Figures 1(b) and 1(c)), indicating that anisomycin promoted HCC cell death partly through activating the p38 MAPK pathway. Several studies have reported that p38 can inhibit cancer stem cell properties [48] and increase apoptosis [49, 50] in cancer cell. Therefore, the expression levels of the cell stemness-related proteins (c-Myc, CD133, Nanog, CD24, and EpCAM) and the apoptosis-related proteins (cleaved caspase-3, Bcl-2, and Bax) were also analyzed in HCC cells during anisomycin treatment, and the changes in protein expression were consistent with the phenotypic changes above (Supplementary Figures 2A-D). To explore whether activation of the p38 pathway regulates ferroptosis in HCC cells, we used C11-BODIPY staining to examine the accumulation of lipid reactive oxygen species (lipid-ROS) and found that anisomycin significantly increased intracellular lipid-ROS, while SB203580 coincubation almost completely rescued the accumulation of lipid-ROS (Figure 1(d)). Similarly, ferroptosis-related proteins were also analyzed by western blotting. The results showed that the expression levels of nuclear factor erythroid 2-related factor 2 (NRF2), solute carrier family 7 member 11 (SLC7A11), and ferritin heavy chain 1 (FTH1) were downregulated by 24 h anisomycin treatment, and the expression levels of these proteins were recovered by SB203580 (Figure 1(e)). In general, anisomycin-induced p38 MAPK activation was cytotoxic to HCC cells, and ferroptosis may be involved in this cytotoxic effect.

### 3.2. Anisomycin Inhibited HCC Cell Migration through the p38 MAPK Signaling Pathway

**Figure 2:**
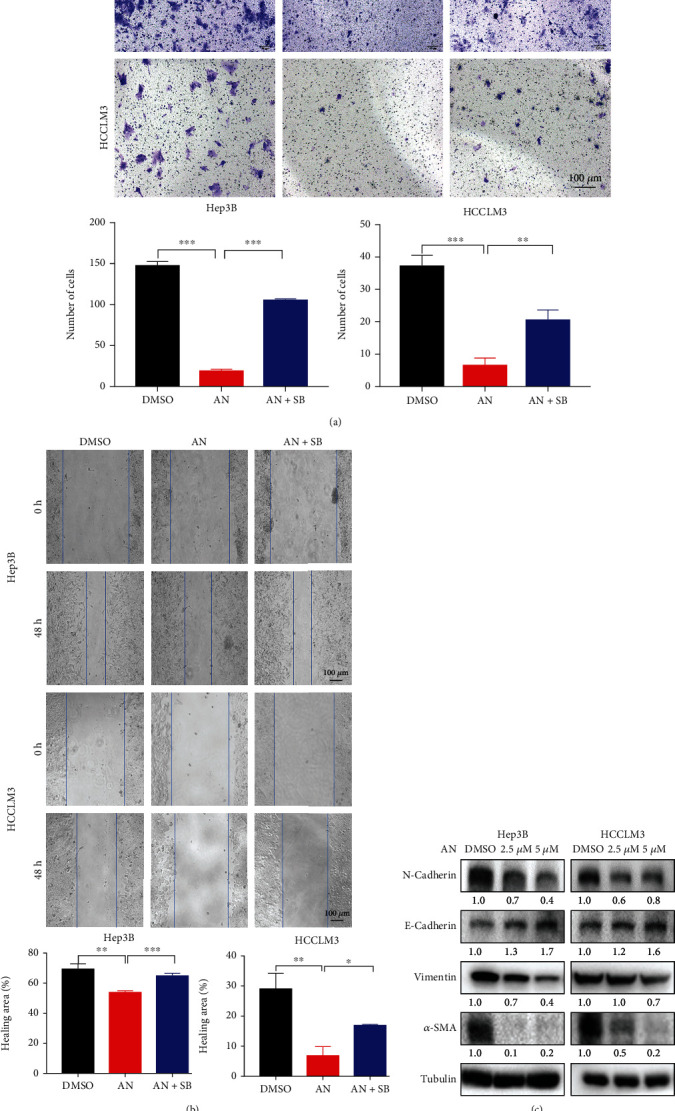
Anisomycin (AN) suppressed HCC cell migration through the p38 MAPK pathway. AN suppressed HCC cell migration (a) and healing ability (b). The effect was evaluated by transwell and wound-healing assay. SB203580 (SB) was used to rescue cells from AN treatment. *N* = 3; ^∗^*P* < 0.05, ^∗∗^*P* < 0.01, and ^∗∗∗^*P* < 0.001. (c) The expression levels of epithelial-mesenchymal transition-related proteins in AN treatment (24 h).

Cell migration also plays an important role in the steps of tumor progression [51]. Transwell and wound-healing assays were performed to assess whether anisomycin could alter the migration ability of HCC cells. The results showed that anisomycin significantly inhibited HCC cell migration, while SB203580 coincubation partially alleviated inhibition of HCC cell migration (Figures 2(a) and 2(b)).

The epithelial-mesenchymal transition (EMT) is a process that accelerates HCC cell migration [51]. EMT protein markers (N-cadherin, E-cadherin, vimentin, and *α*-SMA) were detected during anisomycin treatment by western blotting. After 24 h treatment of anisomycin, E-cadherin expression in HCC cells increased, while N-cadherin, vimentin, and *α*-SMA were decreased (Figure 2(c) and Supplementary Figure 3). The results indicated that anisomycin-activated p38 may inhibit HCC cell migration by inhibiting EMT.

### 3.3. Anisomycin-Activated p38 MAPK Promoted H3S10 Phosphorylation via Colocalization

**Figure 3:**
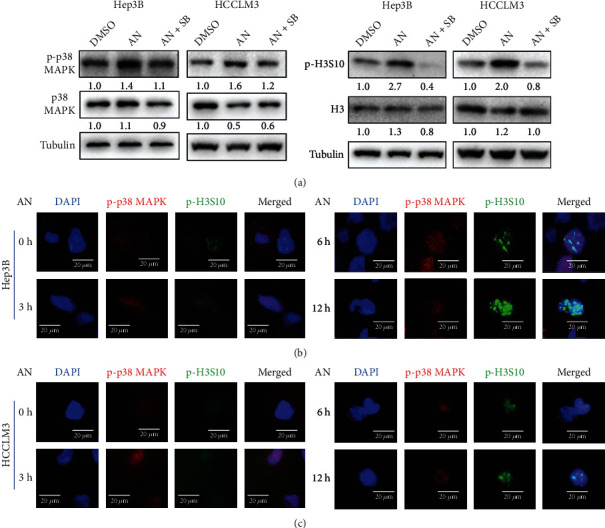
Anisomycin (AN) increased phosphorylation of p38 MAPK and H3S10 in HCC cells. (a) Western blotting analysis of p38 MAPK, p-p38 MAPK, H3, and p-H3S10 in HCC cells after a 24 h DMSO, AN, or AN+SB203580 (SB) treatment. Immunofluorescence costained for p-p38 MAPK and p-H3S10 showed their expression levels and cellular localization at different time points of AN treatment in Hep3B (b) and HCCLM3 (c).

Researchers had found that activation of p38 MAPK could promote histone H3 phosphorylation in Ser10 or Ser28 [22–24]. In our study, the results of the western blotting showed that p-p38 MAPK and p-H3S10 were upregulated after 24 h anisomycin treatment (Figure 3(a)), while the expression level of p-H3S28 was not obviously upregulated (Supplementary Figure 4), suggesting that anisomycin-activated p38 MAPK induced the phosphorylation of H3S10, rather than of H3S28. In Figure 3(a), we can also see that coincubation of SB203580 reduced the activation of p38 MAPK as well as the phosphorylation of histone H3S10, indicating that anisomycin may induce histone H3S10 phosphorylation in HCC cells through p38 MAPK pathway. Then, we costained p-p38 MAPK and p-H3S10 to show their expression levels and cellular localization in Hep3B and HCCLM3 at different time points of anisomycin treatment. Immunofluorescence results showed that p38 MAPK was activated after anisomycin treatment and subsequently promoted histone H3S10 phosphorylation (Figures 3(b) and 3(c)). After 12 h of anisomycin treatment, histone H3S10 phosphorylation was at a high level, so the following experiments were mostly performed at 12 h. Furthermore, phosphorylated H3S10 was colocalized with p-p38 MAPK in HCC cells treated with anisomycin, suggesting that anisomycin-induced p-p38 MAPK may phosphorylate histone H3S10 through colocalization.

### 3.4. Phosphorylated H3S10 Was Enriched in Ferroptosis-Related Gene Promoters

**Figure 4:**
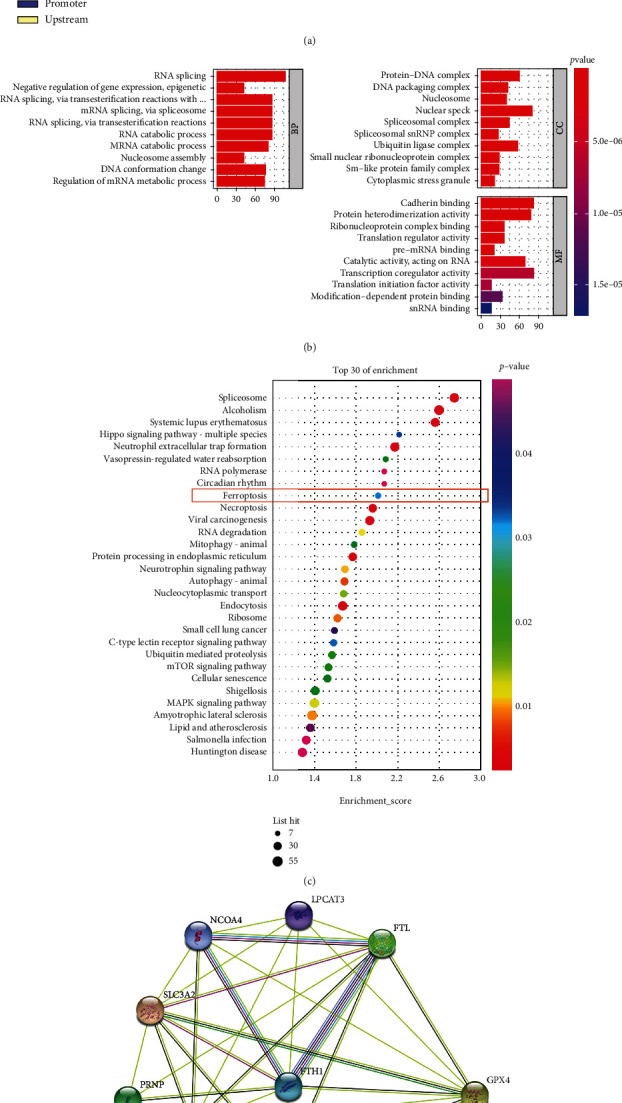
Anisomycin-induced phosphorylation of H3S10 was associated with transcriptional activation of ferroptosis-related genes. (a) Representative graphs of chromatin immunoprecipitation- (ChIP-) sequencing results. (b) Gene Ontology (GO) analyses of differentially enriched genes. BP: biological processes; CC: cell components; MF: molecular functions. (c) Kyoto Encyclopedia of Genes and Genomes (KEGG) results ranked by degree of enrichment. (d) Protein-protein interactions of ferroptosis-related genes enriched in the term of ferroptosis in (c) (NCOA4, SLC3A2, LPCAT3, PRNP, FTL, FTH1, SLC40A1, GCLC, and GPX4).

To elucidate the role of phosphorylated H3S10 in anisomycin-induced HCC cell death, ChIP-sequencing was performed in HCCLM3 cells treated with DMSO or anisomycin (Figure 4(a)) for 12 h. Using a *P* value threshold of 10^−3^, 2445 differentially enriched regions for peak promoter under anisomycin treatment were identified. GO and KEGG were performed to find the main functions and pathways of the genes corresponding to the differentially enriched promoters. The GO results showed differentially enriched biological processes, cell components, and molecular functions (Figure 4(b)), while the KEGG results revealed the differentially enriched pathways (Figure 4(c)). Since we wanted to know how histone H3S10 is involved in anisomycin-induced HCC cell death, we focused on pathways associated with cell death. The enrichment degree of ferroptosis ranked highest in these cell death-related pathways, leading to the conclusion that p-H3S10 may be involved in the regulation of anisomycin-induced ferroptosis in HCC cells. Nine enriched genes were included in the ferroptosis pathway. The PPI plot showed the connections among the nine proteins (Figure 4(d)).

### 3.5. Anisomycin Promoted Ferroptosis of HCC Cells through the p38 MAPK Signaling Pathway

**Figure 5:**
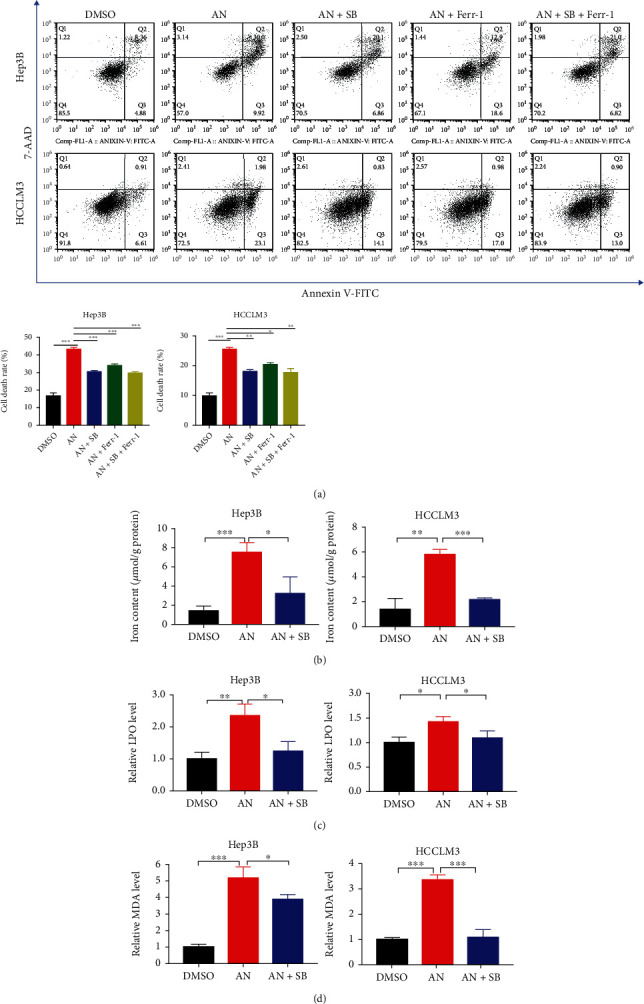
Ferroptosis of HCC cells was stimulated by anisomycin (AN) through the p38 MAPK signaling pathway. (a) Cell death was measured by flow cytometry. Ferrostatin-1 (Ferr-1) and SB203580 (SB) were used to inhibit ferroptosis, thus rescuing cells. (b–d) The assays showed that the content of intracellular iron, lipid peroxidation (LPO), and malondialdehyde (MDA) in HCC cells increased after AN treatment (2.5 *μ*M for Hep3B or 5 *μ*M for HCCLM3). *N* = 3; ^∗^*P* < 0.05, ^∗∗^*P* < 0.01, and ^∗∗∗^*P* < 0.001.

To confirm whether anisomycin induces ferroptosis in HCC cells, a series of experiments were conducted. First, a specific ferroptosis inhibitor, ferrostatin-1, was used in cell treatment to see if it could rescue HCC cells from anisomycin-induced cell death. The results showed that anisomycin-induced cell death was partially inhibited by ferrostatin-1 at both 12 h (Supplementary Figure 5) and 24 h (Figure 5(a)), revealing that ferroptosis was involved in anisomycin-induced HCC cell death. In Figure 5(a), anisomycin + SB203580 + ferrostatin-1 is significantly closer to DMSO than anisomycin + ferrostatin-1; it probably means that p38 induced ferroptosis and other forms of cell death together in anisomycin treatment. To further understand anisomycin-induced ferroptosis, we measured major hallmark features of ferroptosis, such as abnormal accumulation of iron and elevation of lipid peroxidation (LPO) and malondialdehyde (MDA). The iron assay showed that iron increased in HCC cells after being treated with anisomycin and that the increase in iron was suppressed by SB203580 (Figure 5(b)). Similar trends were also observed for the content of LPO and MDA (Figures 5(c) and 5(d)). In general, anisomycin could induce ferroptosis through the p38 MAPK signaling pathway in HCC cells.

### 3.6. NCOA4 Participated in Anisomycin-Induced Ferroptosis in HCC Cells

**Figure 6:**
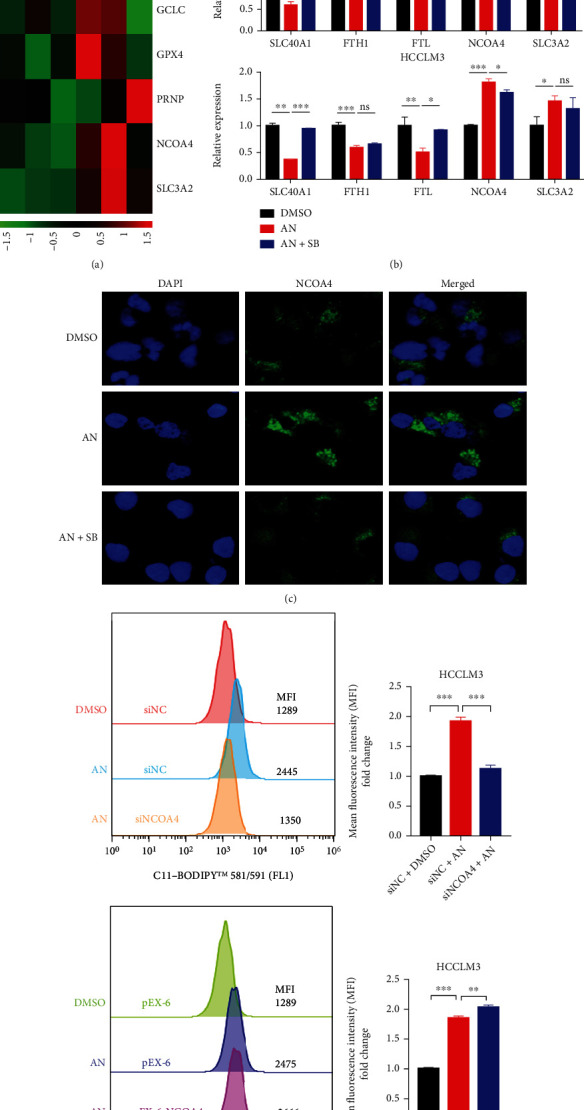
NCOA4 was upregulated in anisomycin- (AN-) induced ferroptosis. (a) The heatmap based on qRT-PCR indicated changes in ferroptosis-related genes in HCCLM3 after treatment with AN for 12 h. In the inhibitor group, cells were pretreated with SB203580 (SB) for 1 h before being treated with anisomycin (2.5 *μ*M for Hep3B or 5 *μ*M for HCCLM3) for 12 h. (b) The qRT-PCR results showed the mRNA expression levels of five ferroptosis-related genes (SLC40A1, FTH1, FTL, NCOA4, and SLC3A2) in different groups of Hep3B and HCCLM3 cells. *N* = 3; ^∗^*P* < 0.05, ^∗∗^*P* < 0.01, and ^∗∗∗^*P* < 0.001. (c) The protein level of NCOA4 was detected in HCC cells after AN treatment by immunofluorescence. (d) The accumulation of lipid-ROS was detected in NOCA4 knockdown or negative control (NC) HCCLM3 after 12 h AN treatment (left). The accumulation of lipid-ROS was detected in NOCA4 cDNA or pEX-6 carrier transfected HCCLM3 after 12 h AN treatment (right).

To further elucidate the potential mechanism of how phosphorylated H3S10 contributed to p38-ferroptosis axis in HCC cells, the mRNA expression levels of ferroptosis-related genes enriched in ChIP-sequencing were further analyzed by RT-PCR and immunofluorescence. Heatmap shows the stable downregulation of solute carrier family 40 member 1 (SLC40A1), ferritin heavy chain 1 (FTH1) and ferritin light chain (FTL), and stable upregulation of nuclear receptor coactivator 4 (NCOA4) and solute carrier family 3 member 2 (SLC3A2) after anisomycin treatment (Figure 6(a)). The recovery of mRNA expression levels of these five genes was further investigated in the SB203580 coincubation group in Hep3B and HCCLM3 (Figure 6(b)). The expression level of NCOA4 was upregulated in both Hep3B and HCCLM3 cells treated with anisomycin and decreased when SB203580 was coincubated. The SLC40A1 results were exactly the opposite. Many previous studies have shown that phosphorylated histone H3S10 always promotes transcription activation [25, 52, 53]. It was speculated that NCOA4 was the key member, upregulated by the p38 MAPK pathway and phosphorylated H3S10, contributing to anisomycin-induced ferroptosis. The immunofluorescence results confirmed that the protein level of NCOA4 was upregulated in HCC cells soon after anisomycin treatment, and SB203580 alleviated the upregulation of NCOA4 (Figure 6(c) and Supplementary Figure 6A). Our findings suggested that anisomycin-induced activation of p38 MAPK may upregulate NCOA4 by phosphorylating H3S10, thus promoting ferroptosis in HCC cells. To illustrate the important role of NCOA4 in anisomycin-induced ferroptosis, we performed NCOA4 knockdown and overexpression in HCC cells and measured the accumulation of lipid reactive oxygen species by flow cytometry after C11-BODIPY staining to infer the effect of NCOA4 on ferroptosis. We found that anisomycin-induced lipid-ROS in HCC cells was almost completely abolished by NCOA4 knockdown, while NCOA4 overexpression slightly increased anisomycin-induced accumulation of lipid-ROS in HCC cells (Figure 6(d) and Supplementary Figure 6B). RT-PCR was used to test NCOA4 knockdown and overexpression in HCC cells (Supplementary Figure 6C). In conclusion, NCOA4 played an important role in anisomycin-induced ferroptosis in HCC cells.

## 4. Discussion

Ferroptosis is emerging as a potential mechanism for suppressing tumor growth because it has been shown to accelerate cell death in some malignancies [54, 55]. The studies of Li et al. [56] and Hattori et al. [44] linked the p38 MAPK signaling pathway to ferroptosis; however, the underlying mechanism of how p38 regulates ferroptosis remains unclear. Since the transcriptional expression of various genes changes during ferroptosis, we speculated that histone modifications may be involved in the regulation of ferroptosis. Many previous studies have shown that activated p38 MAPK could increase the level of p-H3S10 and activate gene transcription by influencing the conformation of chromatin or/and DNA accessibility [27, 28]. Here, we verified the relationship between p38 MAPK and histone H3 modification in HCC and found that anisomycin-activated p38 MAPK was able to activate the level of p-H3S10 by colocalization. The genes regulated by P-H3S10 are involved in a variety of biological processes and pathways. We identified these genes using ChIP-sequencing technology and found that these genes were significantly enriched in ferroptosis by KEGG enrichment analysis. Therefore, anisomycin promoted ferroptosis of HCC cells through the p38 MAPK signaling pathway, as a consequence of being mediated by histone H3S10 phosphorylation.

**Figure 7:**
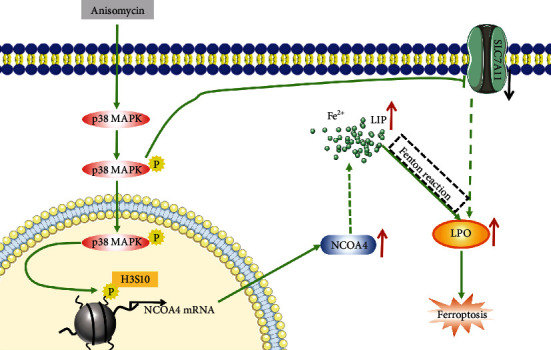
Schematic diagram of anisomycin-induced ferroptosis in HCC cells.

Our study revealed that anisomycin could induce HCC cell death, which was partially reversed by the p38 MAPK inhibitor (SB203580) and the ferroptosis inhibitor (ferrostatin-1). Anisomycin activated p38 MAPK and then promoted H3S10 phosphorylation by colocalization. Many genes are transcriptionally activated by p-H3S10, including NCOA4, which is one of the drivers of ferroptosis [43, 57]. NCOA4, as a cargo receptor that recruits FTH1 to autophagosomes for lysosomal degradation, could lead to ferritinophagy and iron release and then increases the intracellular labile iron pool (LIP) [58, 59]. The alteration of iron in HCC cells directly increased LPO through the Fenton reaction, leading to ferroptosis of HCC cells [60]. Similarly, Yang et al. [61] reported that p38 activation was involved in upregulation of NCOA4 and downregulation of FTH1 in dental pulp stem cells, which was consistent with our results. In our study, FTH1 was decreased by anisomycin at both protein and RNA level in Figures 1(e) and 6(b). However, coincubation with SB203580 resulted in some recovery of FTH1 protein levels (Figure 1(e)), but no significant recovery in FTH1 RNA levels (Figure 6(b)). We already know that SB203580 coincubation reduced NCOA4, so we believed that the recovery of FTH1 in protein level was mainly caused by the reduction of NCOA4, which in turn reduced its recruitment of FTH1 to autophagosomes [58, 59] and ultimately lead to reduced FTH1 degradation and reduced iron release [38, 39]. Furthermore, as we can see in Figure 5(e), the solute carrier family 7 member 11 (SLC7A11) was negatively regulated by anisomycin-activated p38 MAPK. However, the protein level of the key molecule of the classical ferroptosis pathway, glutathione peroxidase 4 (GPX4), did not show significant downregulation in HCC cells after anisomycin treatment (Supplementary Figure 7). Therefore, anisomycin-reducing SLC7A11 may be involved in the regulation of ferroptosis through a non-GPX4 inactivation manner. In addition, SLC7A11 functioned to import cystine for glutathione biosynthesis and ferroptosis defense, and NRF2 positively regulated SLC7A11 transcription [62], both of which are ferroptosis suppressors [63, 64]. The study by Wang et al. [65] reported that activation of p38 MAPK could negatively regulate the expression of SLC7A11 in endometrial cancer cells. In summary, anisomycin could induce HCC cell ferroptosis by increasing LPO and LIP (Figure 7).

By detecting intracellular iron, LPO, and MDA, our study demonstrated that anisomycin could induce ferroptosis in HCC cells. The underlying mechanism of how anisomycin induces ferroptosis was also revealed, which identified anisomycin as a novel ferroptosis stimulus distinct from classical ferroptosis inducers, such as erastin, sulfasalazine, and RSL3 [30, 66]. In our study, anisomycin was used to activate p38 MAPK in HCC cells. Nevertheless, anisomycin is also commonly used as an activator of the JNK signaling pathway. Here, we compared the rescue of anisomycin-induced lipid-ROS by SP600125 (an inhibitor of JNK) and SB203580 (Supplementary Figure 8). As we can see, SB203580 almost completely rescued the accumulation of lipid-ROS in HCCLM3. However, the rescue of anisomycin-induced lipid-ROS by SP600125 was limited. Furthermore, in our study, SB203580, an inhibitor of activated p38 MAPK, partially abrogated the anticancer effects of anisomycin in HCC cells. In conclusion, anisomycin promoted ferroptosis in HCC cells mainly by activating p38 MAPK. Considering the role of p38 MAPK in anisomycin cytotoxicity and the result in supplementary figure 1B, we believed that HCC patients with a low p-p38/p38 ratio may respond better to anisomycin treatment.

It is well known that apoptosis, autophagy, and ferroptosis are different forms of cell death, but they also have some connections to drug treatments [67]. Several studies found that autophagy is a positive regulator of ferroptosis [40, 58, 68]. For example, NCOA4 is one of the key molecules that links autophagy and ferroptosis. Furthermore, iron overload participates not only in the induction of noncanonical ferroptosis but also in endogenous and exogenous apoptosis [34]. Recently, research by Chang et al. [68] revealed that heteronemin could simultaneously induce HCC cell apoptosis and ferroptosis. Besides, HMGB1 has been found to play a vital role in apoptosis, ferroptosis, and autophagy in leukemia cells [32]. From our results, we found that p38 MAPK promoted apoptosis and ferroptosis of HCC cells, making it an ideal target for HCC treatment. Several studies have consistently shown that p38 MAPK was involved in the antiproliferative, apoptotic, and inhibitory effects of EMT in HCC cells [49, 69, 70] and other cancer cells [71–73]. Ras may be involved in the inhibition of HCC cell migration by p38 MAPK pathway, as MAPK provides negative feedback to the Ras activity, which regulates cancer cell migration through PI3K/mTORC2/AKT pathway [74]. Moreover, this mechanism of cell migration is also conserved from 2D culture to 3D organoid [75]. Of course, ferroptosis also has an impact on EMT [76]. The mechanisms involved need to be elucidated by more researchers conducting dedicated studies. In Figure 5(c), there were also necroptosis, autophagy, mitophagy, and cell senescence in the enriched pathways, which suggested that anisomycin-activated p38 MAPK may stimulate HCC cell death through mixed types of cell death. In general, the predominant mode of cell death detected during drug treatment may be related to the concentration of the drug or the duration of treatment [58].

There were also some limitations of our study. First, the function of NCOA4 and FTH1 in ferroptosis was derived from other studies, and no additional experimental validation was performed in our study. Furthermore, how SLC7A11 regulates ferroptosis through a pathway other than GPX4 inactivation in HCC requires further investigation. In addition, the rescue of anisomycin-induced cell death by SB203580 was limited, because anisomycin (AN in figure) is a multifunctional drug that kills cells through multiple targets [77–81]. Moreover, several studies demonstrated that SB203580 could inhibit AKT, and the inhibition of AKT was involved in the induction of ferroptosis and apoptosis [82–84]. We cannot definitively rule out their roles in anisomycin-induced ferroptosis, but we can identify a major role for p38 MAPK. Finally, we will address these limitations to further elucidate the mechanisms of anisomycin-induced HCC cell death in future studies.

In conclusion, anisomycin was confirmed to induce HCC cell ferroptosis through p38 MAPK signaling pathway, and H3S10 phosphorylation may be involved. NCOA4 was revealed to be a key member of p38 MAPK-induced ferroptosis. Furthermore, p38 MAPK may be a worthy target for HCC treatment due to its involvement in various types of cell death.

## Data Availability

The data used to support the findings of this study are available from the corresponding authors upon request.

## Supplementary Materials

Supplementary Materials Supplementary Table 1: the primers used in this study. Supplementary Figure 1: expression levels of p38 MAPK and p-p38 MAPK in 6 HCC cell lines and their inhibition rates at different concentrations of anisomycin for 24 h. Supplementary Figure 2: the expression levels of stemness-related proteins and apoptosis-related proteins. Supplementary Figure 3: the expression levels of epithelial-mesenchymal transition-related proteins in HCC cells after 24 h DMSO, anisomycin (AN), or AN+SB203580 (SB) treatment. Supplementary Figure 4: the protein expression level of p-H3S28 in Hep3B and HCCLM3 after 24 h anisomycin (AN) treatment. Supplementary Figure 5: cell death rate detection was performed on cells treated with DMSO, anisomycin (AN), or AN+ ferrostatin-1 (AN+Ferr-1) for 12 h by flow cytometry. Supplementary Figure 6: NCOA4 played an important role in the p38-ferroptosis axis. Supplementary Figure 7: the protein expression levels of GPX4 in Hep3B and HCCLM3 cells. SB203580 (SB) was used to rescue cells from anisomycin (AN) treatment. Supplementary Figure 8: the rescue of anisomycin-induced lipid-ROS by SB203580 and SP600125 in HCCLM3 cells after 24 h treatment or coincubation.
