# Supplementary material for: Modulation of the p38 MAPK Pathway by Anisomycin Promotes Ferroptosis of Hepatocellular Carcinoma through Phosphorylation of H3S10
Source: Oxid Med Cell Longev. 2022 Nov 24;2022:6986445. doi: 10.1155/2022/6986445 (PMC9715334; doi:10.1155/2022/6986445)
Supplement: Supplementary Materials — Supplementary Table 1: the primers used in this study. Supplementary Figure 1: expression levels of p38 MAPK and p-p38 MAPK in 6 HCC cell lines and their inhibition rates at different concentrations of anisomycin for 24 h. Supplementary Figure 2: the expression levels of stemness-related proteins and apoptosis-related proteins. Supplementary Figure 3: the expression levels of epithelial-mesenchymal transition-related proteins in HCC cells after 24 h DMSO, anisomycin (AN), or AN+SB203580 (SB) treatment. Supplementary Figure 4: the protein expression level of p-H3S28 in Hep3B and HCCLM3 after 24 h anisomycin (AN) treatment. Supplementary Figure 5: cell death rate detection was performed on cells treated with DMSO, anisomycin (AN), or AN+ ferrostatin-1 (AN+Ferr-1) for 12 h by flow cytometry. Supplementary Figure 6: NCOA4 played an important role in the p38-ferroptosis axis. Supplementary Figure 7: the protein expression levels of GPX4 in Hep3B and HCCLM3 cells. SB203580 (SB) was used to rescue cells from anisomycin (AN) treatment. Supplementary Figure 8: the rescue of anisomycin-induced lipid-ROS by SB203580 and SP600125 in HCCLM3 cells after 24 h treatment or coincubation. [file 6986445.f1.docx]

**Supplementary Table 1**

The primers used in this study.

| Gene | Forward primer (5' -> 3') | Reverse primer (5' -> 3') |
| --- | --- | --- |
| SLC40A1 | TGGATGGGTTCTCACTTCCTG | GTCAATCCTTCGTATTGTGGCAT |
| FTH1 | CGAGGTGGCCGAATCTTCC | GTTTGTGCAGTTCCAGTAGTGA |
| FTL | CAGCCTGGTCAATTTGTACCT | GCCAATTCGCGGAAGAAGTG |
| LPCAT3 | GGAGCTGAGCCTTAACAAGTT | CAAAGCAAAGGGGTAACCCAG |
| GCLC | GGCACAAGGACGTTCTCAAGT | CAGACAGGACCAACCGGAC |
| PRNP | AGTCAGTGGAACAAGCCGAG | CTGCCGAAATGTATGATGGGC |
| GPX4 | GAGGCAAGACCGAAGTAAACTAC | CCGAACTGGTTACACGGGAA |
| NCOA4 | ACAGTTGCATAAGCCGTCACC | TGAGCCTGCTGTTGAAGTGTC |
| SLC3A2 | TGAATGAGTTAGAGCCCGAGA | GTCTTCCGCCACCTTGATCTT |
| ACTB | TTGTTACAGGAAGTCCCTTGCC | ATGCTATCACCTCCCCTGTGTG |

**Supplementary Figure 1**


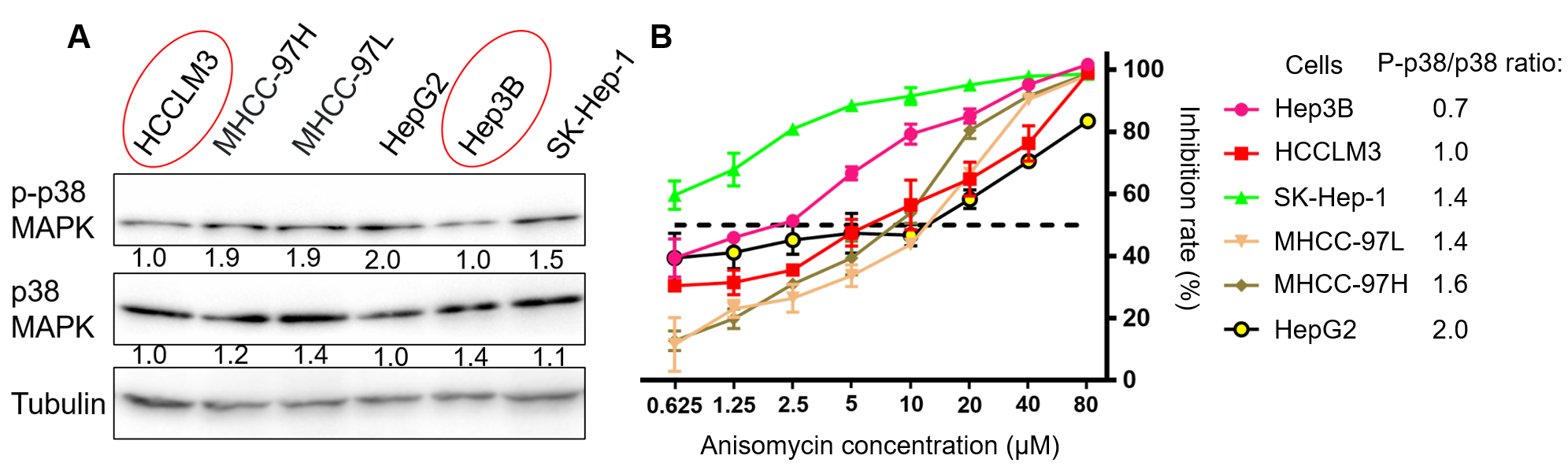


Figure S1. Expression levels of p38 MAPK and p-p38 MAPK in 6 HCC cell lines and their inhibition rates at different concentrations of anisomycin for 24h. **(A)** The expression levels of p38 MAPK and p-p38 MAPK in different HCC cell lines. **(B)** The inhibition rate of three low p-p38/p38 ratio cells (Hep3B, HCCLM3 and SK-Hep-1) and three high p-p38/p38 ratio cells (MHCC-97L, MHCC-97H and HepG2) after 24 h treatment with anisomycin**.**

**Supplementary Figure 2**


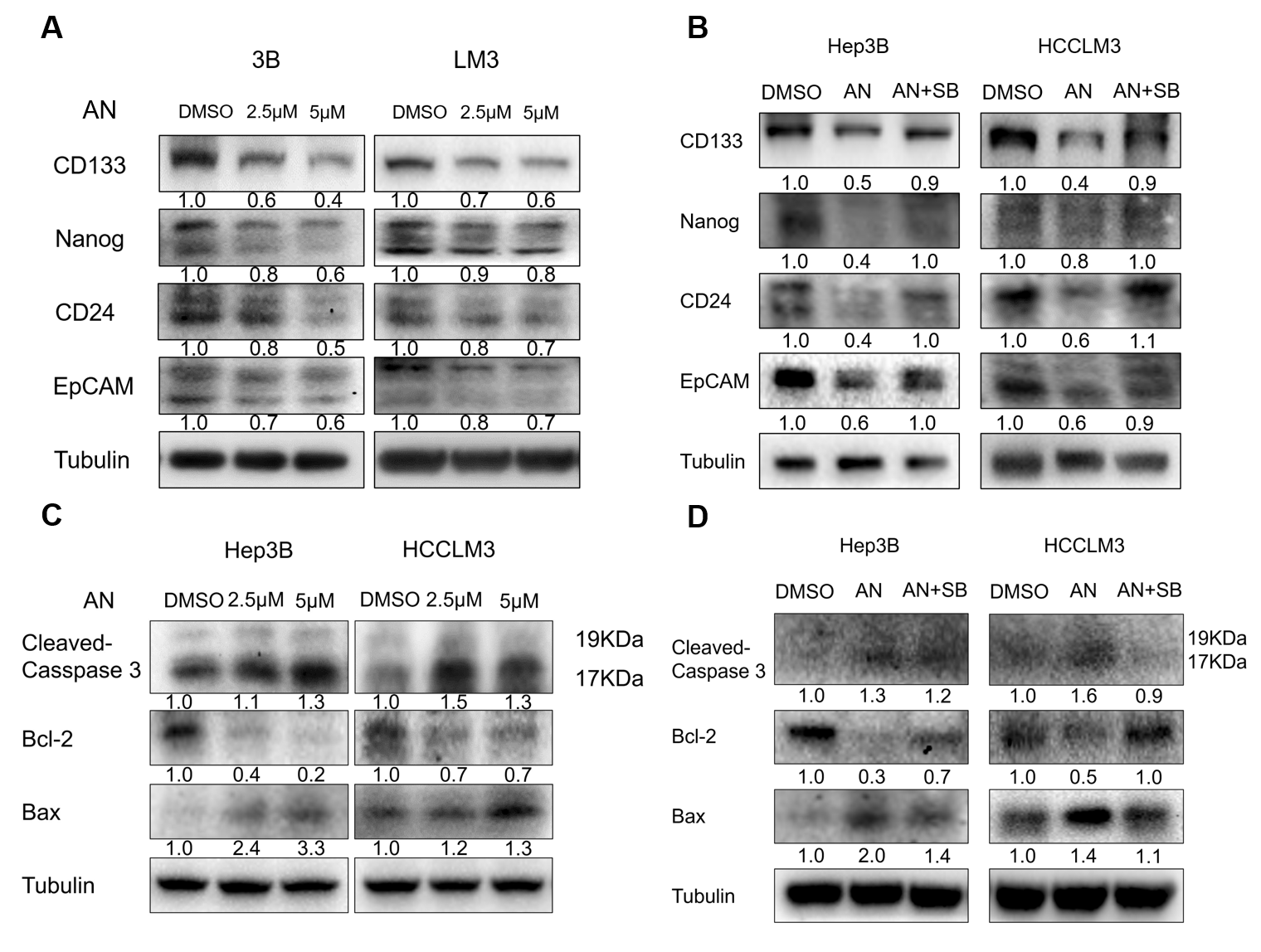


Figure S2. The expression levels of stemness-related proteins and apoptosis-related proteins. Expression levels of cell stemness-related proteins in DMSO, 2.5 μM-anisomycin (2.5 μM-AN), 5 μM-AN samples **(A)** and DMSO, AN, AN+SB203580 (SB) samples **(B)** of HCC cells after 24-h treatment. Expression levels of cell apoptosis -related proteins in DMSO, 2.5 μM-AN, 5 μM-AN samples **(C)** and DMSO, AN, AN+SB203580 (SB) samples **(D)** in HCC cells after 24-h treatment.

**Supplementary Figure 3**


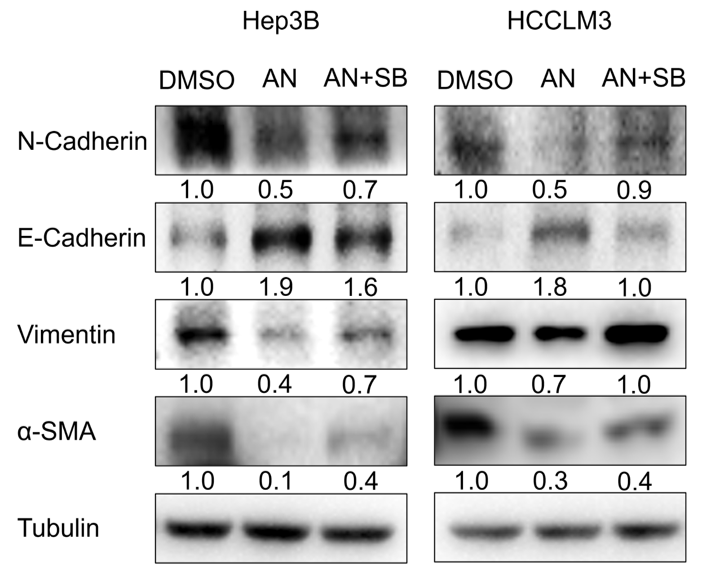


Figure S3. The expression levels of epithelial-mesenchymal transition-related proteins in HCC cells after 24-h DMSO, anisomycin (AN) or AN+SB203580 (SB) treatment.

**Supplementary Figure 4**

**
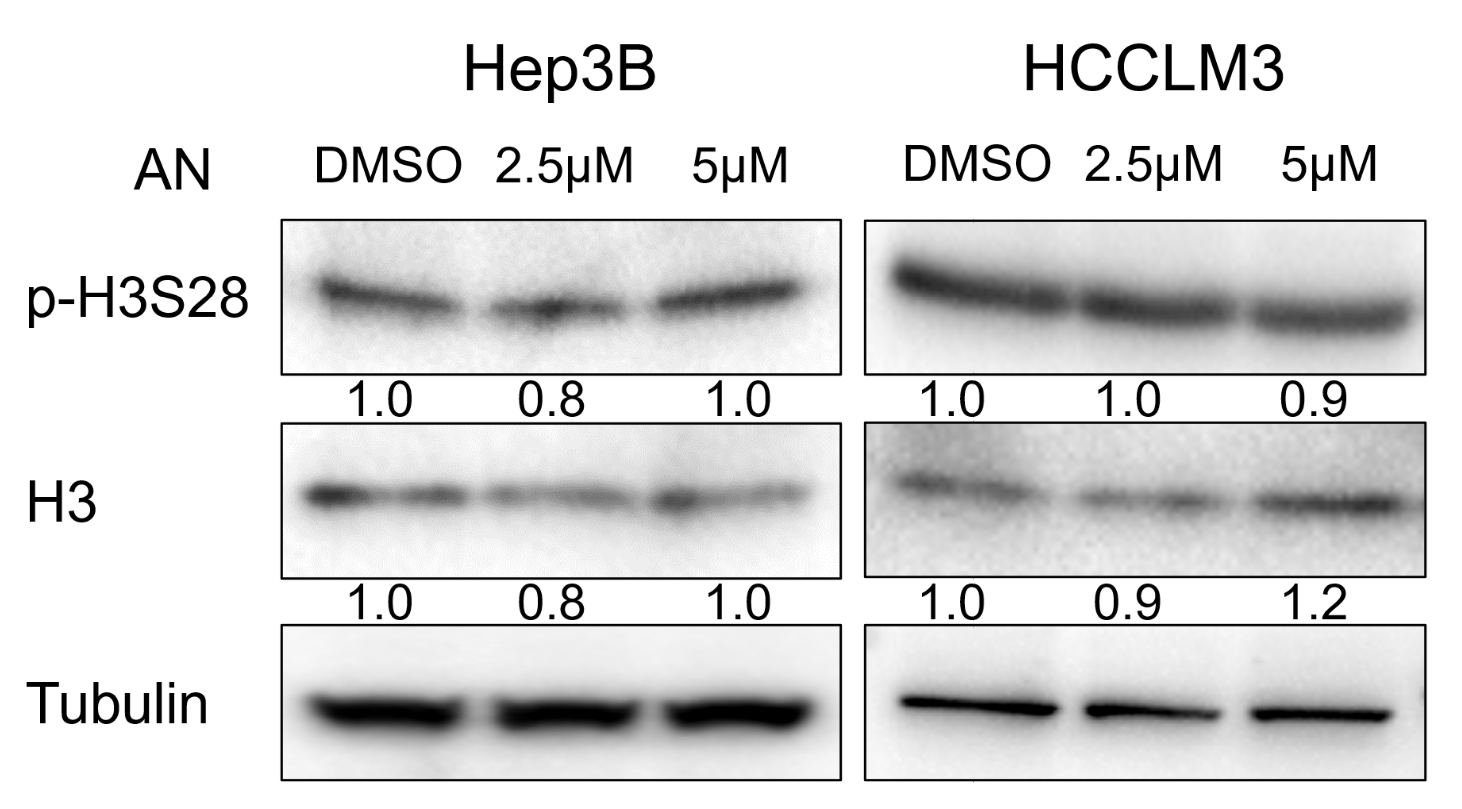
**

Figure S4. The protein expression level of p-H3S28 in Hep3B and HCCLM3 after 24-h anisomycin (AN) treatment.

**Supplementary Figure 5**

**
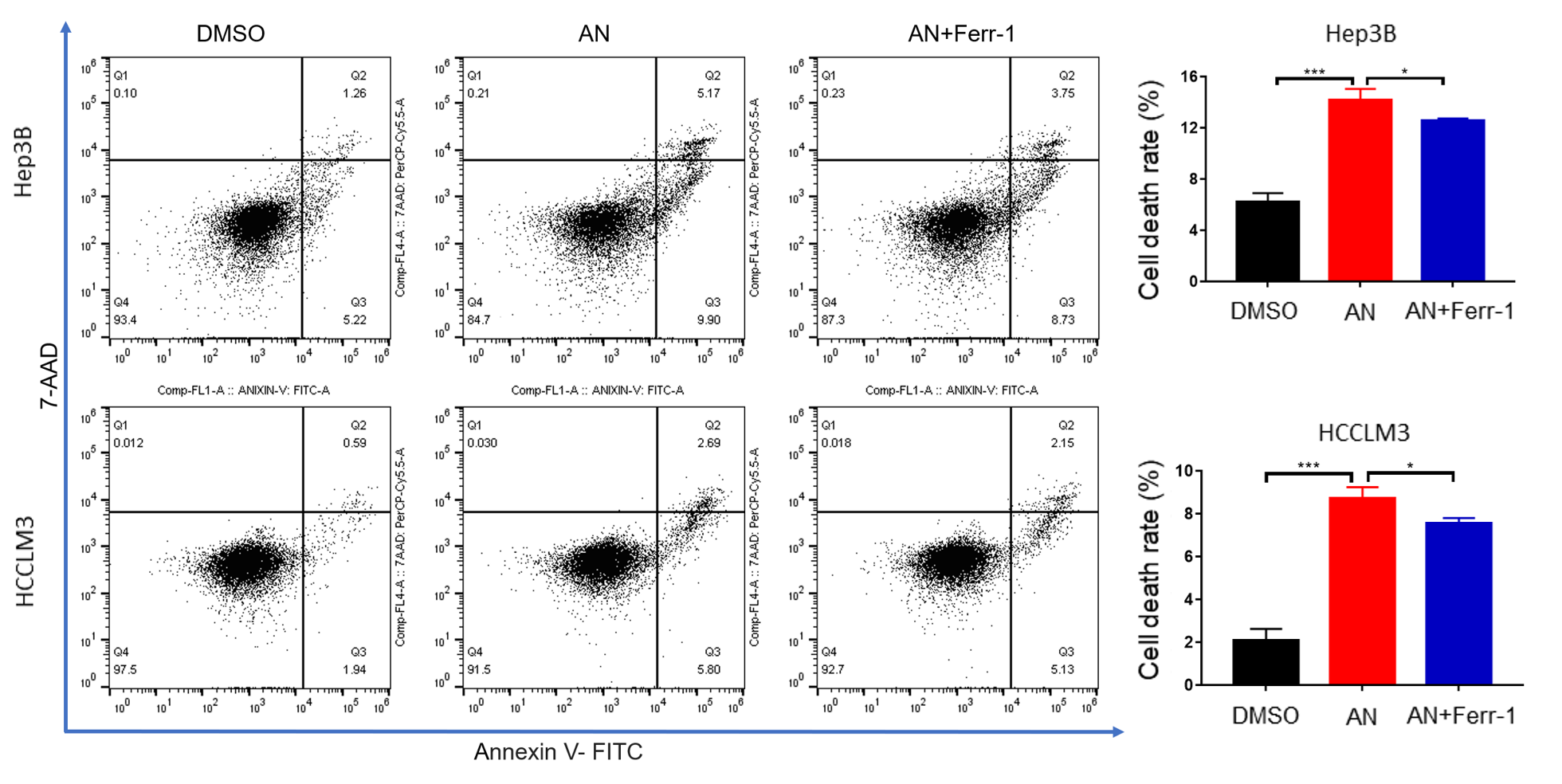
**

Figure S5. Cell death rate detection was performed on cells treated with DMSO, anisomycin (AN), or AN+ Ferrostatin-1 (AN+Ferr-1) for 12h by flow cytometry.

**Supplementary Figure 6**

~~
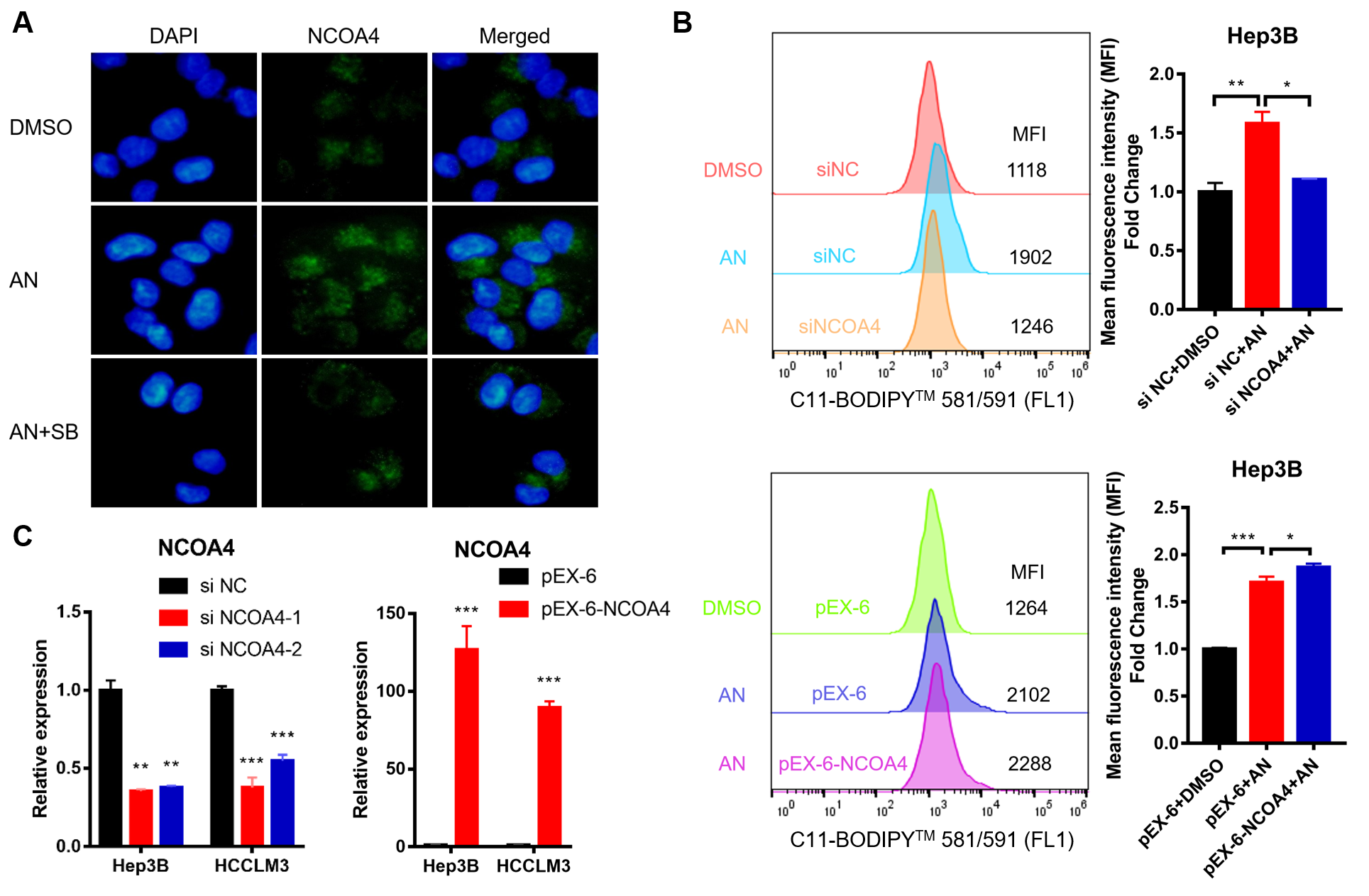
~~

Figure S6. NCOA4 played an important role in the p38-ferroptosis axis. **(A)** Immunofluorescence of NCOA4 in HCCLM3 cells treated with AN. **(B)** The accumulation of lipid-ROS was detected in NOCA4 knockdown or negative control (NC) Hep3B after 12-h AN treatment (Above). The accumulation of lipid-ROS was detected in NOCA4 cDNA or pEX-6 carrier transfected Hep3B after 12-h AN treatment (Below). **(C)** RT-PCR was used to test NCOA4 knockdown and overexpression in Hep3B and HCCLM3.

**Supplementary Figure 7**

**
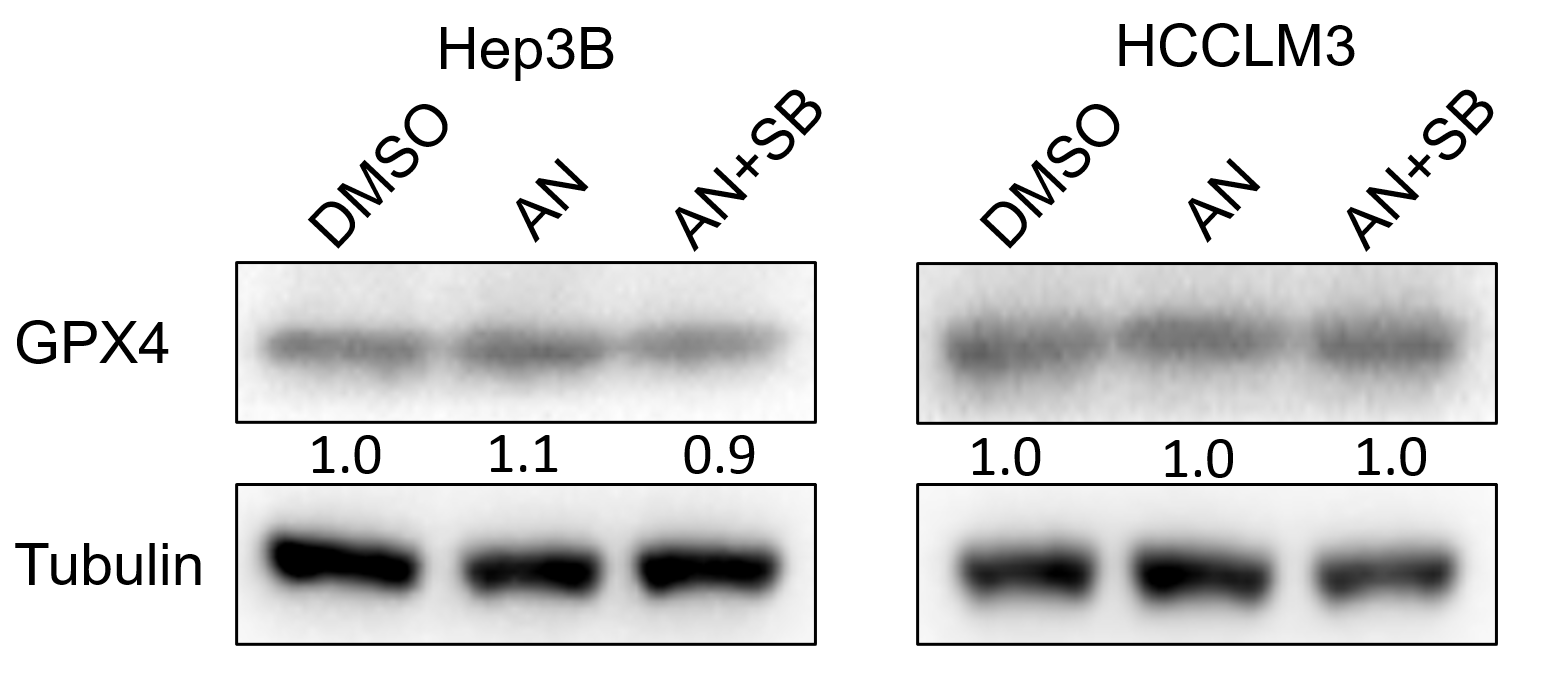
**

Figure S7. The protein expression levels of GPX4 in Hep3B and HCCLM3 cells. SB203580 (SB) was used to rescue cells from anisomycin (AN) treatment.

**Supplementary Figure 8**

**
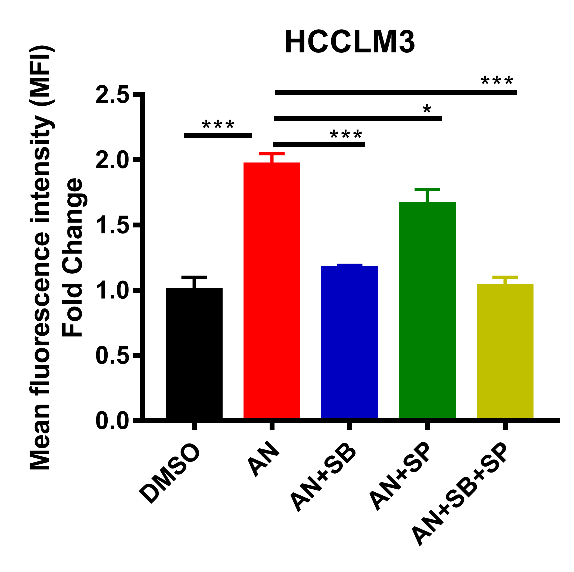
**


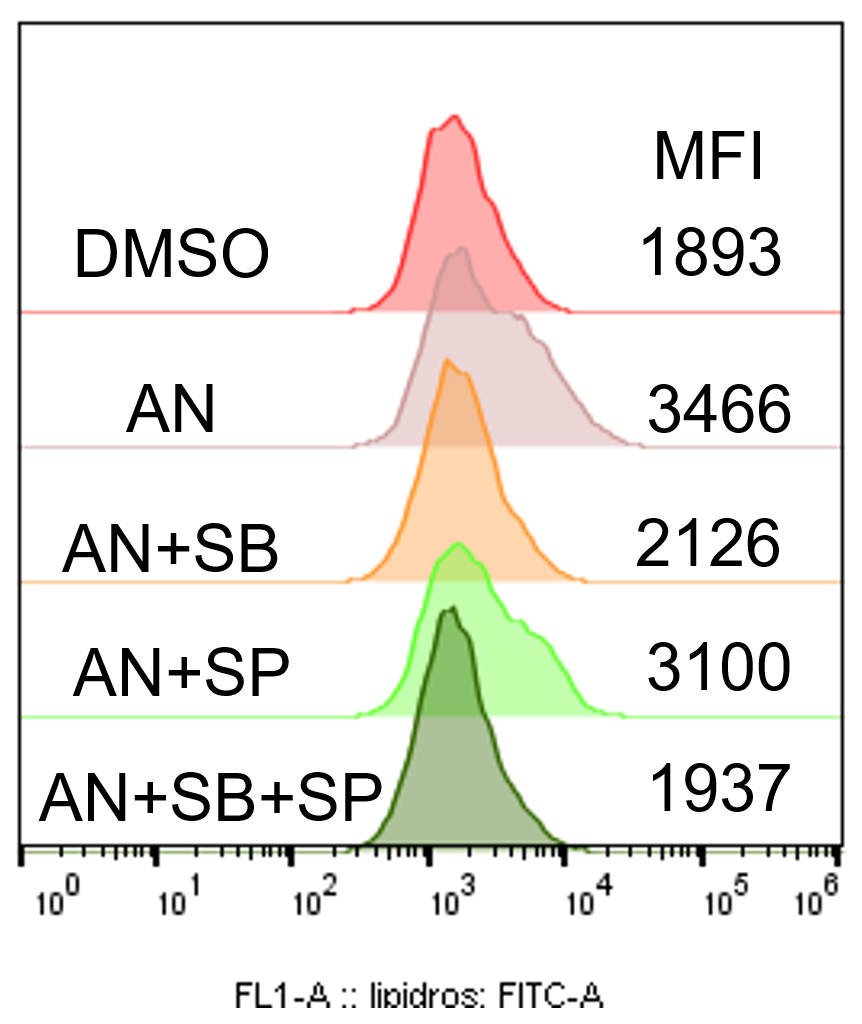


C11-BODIPY^TM^ 581/591 (FL1)

Figure S8. The rescue of anisomycin-induced lipid-ROS by SB203580 and SP600125 in HCCLM3 cells after 24-h treatment or co-incubation.
